# Contribution of the *Pseudomonas fluorescens* MFE01 Type VI Secretion System to Biofilm Formation

**DOI:** 10.1371/journal.pone.0170770

**Published:** 2017-01-23

**Authors:** Mathias Gallique, Victorien Decoin, Corinne Barbey, Thibaut Rosay, Marc G. J. Feuilloley, Nicole Orange, Annabelle Merieau

**Affiliations:** 1 LMSM, Laboratoire de Microbiologie Signaux et Microenvironnement, EA 4312, IUT d'Evreux, Université de Rouen, Normandy University, Evreux, France; 2 Seeds Innovation Protection Research and Environment (SIPRE), Achicourt, France; Centre National de la Recherche Scientifique, Aix-Marseille Université, FRANCE

## Abstract

Type VI secretion systems (T6SSs) are widespread in Gram-negative bacteria, including *Pseudomonas*. These macromolecular machineries inject toxins directly into prokaryotic or eukaryotic prey cells. Hcp proteins are structural components of the extracellular part of this machinery. We recently reported that MFE01, an avirulent strain of *Pseudomonas fluorescens*, possesses at least two *hcp* genes, *hcp1* and *hcp2*, encoding proteins playing important roles in interbacterial interactions. Indeed, *P*. *fluorescens* MFE01 can immobilise and kill diverse bacteria of various origins through the action of the Hcp1 or Hcp2 proteins of the T6SS. We show here that another Hcp protein, Hcp3, is involved in killing prey cells during co-culture on solid medium. Even after the mutation of *hcp1*, *hcp2*, or *hcp3*, MFE01 impaired biofilm formation by MFP05, a *P*. *fluorescens* strain isolated from human skin. These mutations did not reduce *P*. *fluorescens* MFE01 biofilm formation, but the three Hcp proteins were required for the completion of biofilm maturation. Moreover, a mutant with a disruption of one of the unique core component genes, MFE01Δ*tssC*, was unable to produce its own biofilm or inhibit MFP05 biofilm formation. Finally, MFE01 did not produce detectable N-acyl-homoserine lactones for quorum sensing, a phenomenon reported for many other *P*. *fluorescens* strains. Our results suggest a role for the T6SS in communication between bacterial cells, in this strain, under biofilm conditions.

## Introduction

The type VI secretion system (T6SS) is a widespread macromolecular machinery in Gram-negative bacteria [1]. This multiprotein complex delivers effectors into eukaryotic and/or bacterial cells [1–5]. This secretion system has a structure similar to that of the contractile tails of bacteriophages [6,7]. Hcp (hemolysin-coregulated protein) and VgrG (valine-glycine repeat protein G) are structural proteins of this machinery with structural similarities to the gp19 and gp5-gp27 proteins of bacteriophage T4, respectively [8,9]. The structural homologue of the phage tube is built from rings of Hcp hexamers with a tip complex composed of VgrG trimers, Paar protein, and effectors [10–15]. This tube is ejected by the contraction of a tubular sheath consisting of the conserved T6SS-associated cytoplasmic proteins, TssB and TssC [16–19]. Hcp proteins appear to be directly involved in effector recognition [13,20–23], acting in synergy with VgrG proteins. Moreover, many VgrG proteins possessing a variety of effector domains at their C-termini, are effectors as well as structural components [4].

The T6SS is associated with several phenotypes, including biofilm formation [24]. In natural, industrial, and clinical environments, bacteria live predominantly in biofilms [25]. Biofilms act as reservoirs of infection, protecting the bacteria they contain against diverse external aggressions. They contribute to many problems, particularly in hospitals, where they may form on catheters or implants (e.g. hip prostheses) [26]. *Pseudomonas aeruginosa* biofilms also aggravate bacterial infections in a human chronic wound mouse model [27]. The transition between the planktonic and biofilm states is linked to the production of adhesins and extracellular matrix (ECM) [28]. Adhesins, including flagellin, play a crucial role, by fixing the bacteria to the support, allowing biofilm formation to occur [28–30]. The ECM surrounding the bacteria may consist of exopolysaccharides (EPS), proteins, and DNA [31]. EPS act as adhesins on inert and living surfaces, promoting biofilm and microcolony formation. They are also involved in protection against antibacterial compounds [32–34]. In pseudomonads, quorum-sensing regulates the production of extracellular DNA, lectins, and biosurfactants, all of which play a role in biofilm formation [25].

We previously described *P*. *fluorescens* MFE01, a strain that secretes large amounts of Hcp proteins (mainly Hcp2 proteins) into the culture medium [35]. MFE01 is nonvirulent against various eukaryotic cell models, but has antibacterial activity against a wide range of competitor bacteria, including rhizobacteria and clinical bacteria [35]. The Hcp2 protein has been directly implicated in the killing activity of MFE01. Another Hcp protein, Hcp1, is encoded by *hcp1*, mutation of which has pleiotropic effects on the phenotype of MFE01, affecting its mucoidy and motility [36]. However, *hcp1* mutation has no effect on bacterial competition during incubation on solid medium. Moreover, MFE01 and its *hcp2* mutant can sequester a clinical *P*. *fluorescens* strain, MFN1032, under swimming and swarming conditions, whereas the *hcp1* mutant of MFE01 cannot. Hcp1 appears to reduce the motility of prey cells, to facilitate killing by Hcp2 [36].

We carried out a genomic analysis to investigate the genetic determinants of the T6SS of MFE01. This analysis revealed the existence of a unique T6SS core component locus and another orphan *hcp* gene, *hcp3*. We investigated the possible role of the T6SS in MFE01 biofilm formation and bacterial competition. We also studied the T6SS-mediated antibacterial activity of *P*. *fluorescens* MFE01 and biofilm formation by a cutaneous isolate of *P*. *fluorescens* in contact with MFE01.

## Materials and Methods

### Bacterial strains, plasmids and culture conditions

All the strains and plasmids used are listed in Table 1. All bacterial strains were grown in LB (Luria Bertani) medium with shaking (180 rpm). *P*. *fluorescens* strains were grown at 28°C, *Pectobacterium atrosepticum*, *Agrobacterium tumefaciens*, and *Chromobacterium violaceum* were cultured at 25°C, and *Pseudomonas aeruginosa* and *Escherichia coli* strains were grown at 37°C. The medium was supplemented with antibiotics, as appropriate: kanamycin (Km) at 100 μg/mL (*P*. *fluorescens*) or 200 μg/mL (*P*. *aeruginosa*); tetracycline (Tc) at 15 μg/mL; gentamicin (Gt) at 50 μg/mL or 100 μg/mL (*P*. *fluorescens* in liquid and solid media, respectively).

**Table 1:** Plasmids and strains used in this study.

Strains or plasmids	Relevant characteristics	Reference/source
***P*. *fluorescens***		
MFE01	Air isolate, Rif^R^	[35]
MFE01Δ*hcp2*	MFE01 with early stop codon in *hcp2*	[35]
MFE01Δ*hcp2Chcp2*	MFE01Δ*hcp2* with wild type *hcp2* gene reintroduced in its original chromosomal location (previously named MFE01Δ*hcp2*.*R)*	[35]
MFE01Δ*hcp1*	MFE01 with *hcp1* disruption	[36]
MFE01Δ*hcp3*	MFE01 with early stop codon in *hcp3*	This work
MFE01Δ*hcp3+*EV	MFE01Δ*hcp3* with empty pPSV35	This work
MFE01Δ*hcp3+hcp3*	MFE01Δ*hcp3* with pPSV35 containing *hcp3*	This work
MFE01Δ*tssC*	MFE01 with early stop codon in *tssC*	[36]
MFE01Δ*tssC+tssC*	MFE01Δ*tssC* with pPSV35 containing *tssC*	[36]
MFE01Δ*tssC+*EV	MFE01Δ*tssC* with empty pPSV35	This work
MFN1032	Clinical strain	[50]
MFP05	Skin isolate	[52]
MFP05-gfp	MFP05 with pSMC2.1-gfp	[35]
***P*. *aeruginosa H103***	Prototrophic derivative of PAO1	[51]
***Agrobacterium tumefaciens* NTI**	Strain with plasmid pZLR4 carrying *traR* and a *traG*::*lacZ* reporter fusion from pTiC58. ß–galactosidase producer in contact with C6- to C12-N-Acyl-Homoserine-Lactones (AHLs).	[43]
***Pectobacterium atrosepticum 6276***	Isolate from *Solanum tuberosum*, C8-AHLs producer	[44]
***Pectobacterium atrosepticum 6276 ΔexpI***	*Pectobacterium atrosepticum* 6276 with mutation of the N-Acyl-Homoserine-Lactones (AHLs) synthetase gene	[45]
***Chromobacterium violaceum* CV026**	Mini-Tn5 mutant of *Chromobacterium violaceum*. Violacein producer in contact with C4- to C8-AHLs	[42]
***E*.*coli* S17.1**	RP4-2-Tc::Mu, *aph*::Tn7, *recA*, Sm^R^, donor strain for conjugation	[38]
**Vectors**		
pPSV35	*P*. *aeruginosa oriV*, *lacIq*, *mob+*, P*lac*UV5, pUC18 MCS, expression vector, Gm^R^	[39]
pSMC2.1-gfp	Replicative plasmid, Km^R^, *gfp*	[41]
pAKE604	Ap^R^, Km^R^, oriT *lacZ*, *sacB*	[37]

### Construction of the MFE01*Δhcp3* mutant

Gene knockout was performed, as previously described [36], with the suicide plasmid pAKE604 [37]. In-frame *hcp3* disruption, due to the deletion of a central region of about 300 bp, was achieved by PCR with the muta1-hcp3-F and muta2-hcp3-R primers (product of about 650 bp) or the muta3-hcp3-F and muta4-hcp3-R primers (product of about 600 bp) (Table 2). The PCR products obtained corresponded to the upstream and downstream parts of the *hcp3* gene of MFE01, respectively, each carrying an overlapping sequence at the end. The PCR parameters were as follows: 30 cycles with annealing at 61°C, and extension for 45 s. A third PCR was then carried out with the muta1-hcp3-F and muta4-hcp3-R primers. The PCR parameters were as follows: 35 cycles with annealing at 61°C and extension for 1 min 45 s. The resulting disrupted *hcp3* was inserted into the pAKE604 suicide vector digested with the blunt-ended restriction enzyme *Sma*I, to which it was ligated with T4 DNA ligase (NEB). This construct was verified by sequencing and then introduced into *E*. *coli* strain S17-1 [38]. The recombinant plasmid was transferred by biparental mating: the recipient strain MFE01 and the S17-1 strain containing pAKE604Δ*hcp3* were mixed in equal amounts, spotted onto LB agar and incubated at 37°C overnight. The resulting colonies were resuspended in 1 mL sterile saline and 0.1 mL of the cell suspension was spread on LB agar plates supplemented with 50 μg/mL rifampicin (to select MFE01 and kill *E*. *coli* S17-1) and 200 μg/mL kanamycin (to select cells containing the recombinant plasmid), which were then incubated at 28°C for 48 h. The resulting colonies were isolated and plated on LB agar plates supplemented with 10% sucrose to select for a second homologous recombination event. The resulting *hcp3* mutant was verified by DNA sequencing and named MFE01Δ*hcp3*.

**Table 2:** Oligonucleotides used for this study.

Primer name	Primer sequence (54’-3’)
Muta1-hcp3-F	TCGACCACAACCATACAACA
Muta2-hcp3-R	ATACTTCGTGGGTCCAGGTGATAAGTCCACTTCAGCTACCG
Muta3-hcp3-F	ATCACCTGGACCCACGAAGTATCACCATGATCTTGTCTTCGC
Muta4-hcp3-R	TGGATCAGCAAGTCCAGATC
hcp3-EcoRI-F	TAATAAGAATTCACTTCATAACTAAGGAGCCA
hcp3-XbaI-R	TAATAATCTAGAGTCGGTCATGCTCAGTTC

### Insertion of the *hcp3* gene into pPSV35 and the construction of strain MFE01Δ*hcp3*+*hcp3*

The *hcp3* gene was amplified from *P*. *fluorescens* strain MFE01 with the hcp3-EcoRI-F and hcp3-XbaI-R primers (Table 2). The PCR conditions were as follows: 30 cycles with an annealing temperature of 58°C and an extension time of 40 s. The Phusion® High-Fidelity DNA polymerase (NEB) was used. The amplified fragment and the pPSV35 shuttle vector [39] were digested with *Eco*RI and *Xba*I (NEB) to generate cohesive ends, and the *hcp3* gene was inserted into the vector by DNA ligase mediated ligation (NEB). The resulting plasmid, pPSV35-*hcp3*, was used to transform *E*. *coli* Top10® cells by thermal shock. Plasmid DNA was isolated with the GeneJET Plasmid Miniprep Kit (ThermoFisher Scientific) and verified by PCR with primers binding to the plasmid. Fresh MFE01Δ*hcp3* colonies were obtained and washed twice with 1 mL cold sterile water before transformation with 5 μL pPSV35-*hcp3* by electroporation in 1 mm electroporation cells at 1.8 KV for 5 milliseconds (Savant GTF100 Gene Transformer). LB was added and the mixture was incubated at 28°C for 1 h with shaking (180 rpm). Transformed bacteria were then selected by plating on LB agar supplemented with gentamycin and IPTG.

### Biofilm formation

Biofilms derived from pure cultures and co-cultures were grown in three-channel flow cells for continuous culture (channel dimensions, 1 by 4 by 40 mm). The system was assembled and prepared as follows: sterilisation with 1.2% bleach for 4 h, followed by rinsing in sterile saline. Before flow chamber inoculation, LB medium was injected into the system at 28°C. Overnight cultures were centrifuged at 8,000 x *g* for 5 min at room temperature. Pellets were recovered and washed twice in 2 mL sterile saline. For pure-culture biofilm assays, the channels were inoculated with 1 mL pure bacterial suspension at an OD_580_ of 0.1. For co-culture biofilms, MFE01 or mutant suspensions at an OD_580_ of 0.1 were mixed with a bacterial suspension of the competitor (MFP05 strain carrying pSMC2.1-gfp plasmid) at an OD_580_ of 0.1, in a 5:1 ratio. The channel was inoculated by injection of the mixture (1 mL). Bacteria were allowed to attach to the glass surface (microscope coverslip, VWR International, Fontenay sous Bois, France) for 2 h at 28°C under static conditions. A biofilm was then allowed to grow under a constant flow (Wakson Marlow 205S, 2.5 mL/h) of LB medium, with antibiotics at the appropriate concentration if necessary, for 48 h at 28°C. Bacteria were used with or without dilution to an OD_580_ of 0.1 in the MFE01 supernatant, to investigate the potential effects of molecules secreted by *P*. *fluorescens* MFE01 on biofilm formation by *P*. *fluorescens* MFP05. This experiment was carried out as described by Dheilly *et al*., without modification [40].

### Confocal laser scanning microscopy (CLSM) and image analysis

Microscopy was performed with an LSM 710 system (Zeiss, Germany), using a 63x oil immersion objective. Co-culture biofilms were observed by monitoring the GFP fluorescence of competitor MFP05 transformed with pSMC1-2 gfp [41] (excitation wavelength [λ], 488 nm; emission, 510 nm). Biofilm stacks were analysed with COMSTAT software. The calculated parameter was biovolume, defined as the volume of bacteria (in μm^3^) per μm^2^ of glass. In these conditions only MFP05 transformed with pSMC1-2 gfp were detected.

Biovolume of biofilms from pure cultures (MFE01 and derivatives) were observed and analysed with COMSTAT software after 15 min of incubation with 5 μM Syto 9® green fluorescent nucleic acid stain (Life Technologies; excitation at 488 nm and emission at 510 nm). The results are the means of at least five independent experiments.

### Killing assay on solid medium

Killing assays on solid media were performed as described by Decoin *et al*. [35]. In brief, predator and prey bacteria were mixed at a 1:5 ratio unless otherwise stated. Bacteria were spotted on LB plates and incubated at 28°C for 4 h. Bacteria were resuspended, diluted and plated on LB plates containing appropriate antibiotics for the specific selection of predator or prey.

The competitive index was calculated using the equation: (input attacker/input prey)/(output attacker/output prey).

### *In silico* identification of T6SS loci

The genome of *P*. *fluorescens* MFE01 (Merieau, unpublished results) was screened for genes displaying sequence identity to those associated with the T6SS, by NCBI Blast (stand-alone) (http://www.ncbi.nlm.nih.gov/). The sequences for the *tssA* to *tssM* genes of *P*. *fluorescens* strains, available from Pseudomonas.com (http://www.pseudomonas.com), were used as bait in Blast searches to identify homologues in the MFE01 genomic sequence (e-value <10^−3^). The genome was also analysed with the SecReT6 (http://db-mml.sjtu.edu.cn/SecReT6/) “CDeasy” tool. Each putative T6SS ORF was subjected to phyre2 analysis (http://www.sbg.bio.ic.ac.uk/phyre2/).

### N-acyl-homoserine lactone (AHL) detection

*Chromobacterium violaceum* CV026 (CV026) [42] is a mini-Tn5 mutant in which violacein (a purple pigment) production can be restored by incubation with supernatants from the wild-type strain. In CV026, violacein production is inducible by AHL compounds with N-acyl side chains for four to eight carbons (C4-AHL to C8-AHL). This assay was performed by cross-streaking MFE01 against *C*. *violaceum* CV026 on LB plates, which were then incubated for 72 h at 28°C.

*Agrobacterium tumefaciens* NTI [43] was used as a biosensor producing ß-galactosidase (generating a blue colour on X-gal plates) in response to AHLs with N-acyl side chains for six to twelve carbons (C6-AHL to C12-AHL). This assay was performed by cross-streaking MFE01 against *A*. *tumefaciens* NT1 on LB plates supplemented with X-Gal (40 μg/mL), which were then incubated for 72 h at 28°C.

*Pectobacterium atrosepticum 6276* [44] was used as a positive control for the biosensors. It produces C8-AHLs that activate *C*. *violaceum* CV026 and *A*. *tumefaciens* NT1 biosensors. *Pectobacterium atrosepticum 6276 Δexp1*, impaired in C8-AHL production, was included as a negative control [45].

### Statistical analysis

Non-parametric Mann-Whitney tests (two tailed) with GraphPad Prism version 6.0 (La Jolla, CA) were used for statistical analyses. A *p*-value < 0.05 was considered to be statistically significant.

## Results and Discussion

### The MFE01 strain has a single T6SS cluster and at least three *hcp* genes

We previously identified two *hcp* genes, *hcp1* and *hcp2*. The expression of *hcp2* contributes to the killing activity of MFE01 against many Gram-negative bacteria, whereas *hcp1* expression is involved in the immobilisation of prey cells. The competitor population was nevertheless reduced slightly by the double mutant, MFE01*Δhcp1Δhcp2*, suggesting the involvement of another factor in T6SS-mediated MFE01 killing activity [36].

**Fig 1:**
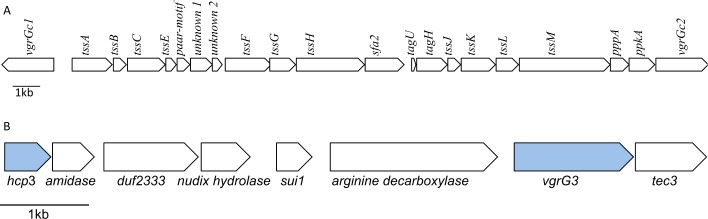
Genomic organisation of the T6SS core component locus and the *hcp3* locus in MFE01. **A. Genomic organisation of the T6SS core component locus in MFE01.** Genes are represented as arrows, indicating the direction of transcription. The sequences of the T6SS core component genes have been deposited in GenBank under the following accession numbers: *vgrGc1*: KX941475, *tssA*: KX941476, *tssB*: KX941477, *tssC*: KX941478, *tssE*: KX941479, *paar-motif*: KX941480, *unkown1*: KX907122, *unknown2*: KX941481, *tssF*: KX941482, *tssG*: KX941483, *tssH*: KX941484, *sfa2*: KX941485, *tagU*: KX941486, *tagH*: KX941487, *tssJ*: KX941488, *tssK*: KX941489, *tssL*: KX941490, *tssM*: KX941491, *pppA*: KX941492, *ppkA*: KX941493, *vgrGc2*: KX941494. **B. Genomic organisation of the *hcp3* locus.** Genes are represented as arrows, indicating the direction of transcription. The sequences of the *hcp3* locus genes have been deposited in GenBank under the following accession numbers: *hcp3*: KX941495, *vgrG3*: KX941496, *arginine-decarboxylase*: KX941497, *sui1*: KX941498, *nudix-hydrolase*: KX941499, *duf2333*: KX941500, *amidase*: KX941501, *tec3*: KX941502.

Exhaustive studies of the MFE01 draft genome confirmed that it contained only one T6SS cluster. All the T6SS core component genes (*tssA* to *tssM*) were observed in classic synteny, except *tssD* genes (or *hcp*), which were distally located from other T6SS core genes, and *tssI* gene (or *vgrG*), which was present as two copies in opposite orientations (*vgrGc1* and *vgrGc2*), on either side of the cluster. This cluster contains six conserved T6SS-associated genes (*paar*, *sfa2*, *tagU*, *tagH*, *pppA*, and *ppkA*) and two predicted ORFs (*unknown1* and *unknown 2*) (Fig 1A). We identified a third orphan *hcp* gene, *hcp3*, during annotation of the MFE01 genome. The *hcp3* gene is co-localised with a *vgrG gene*, *vgrG3*, and several putative ORFs (Fig 1B). The first ORF downstream from *hcp3* encodes a putative N-acetylmuramoyl-L-alanine amidase. N-acetylmuramoyl-L-alanine amidases have been identified as T6SS effectors or Tae (Type 6 amidase effector), and are involved in antibacterial activity [46]. The next gene downstream, *duf2333*, belongs to a conserved but uncharacterised superfamily. The third gene encodes a putative NUDIX hydrolase. Duong-Ly *et al*. showed that a member of the NUDIX hydrolase family from *Streptococcus pneumonia*, UDP-X diphosphatase, hydrolysed UDP-N-acetylmuramic acid and UDP-N-acetylmuramoyl-L-alanine, two substrates for peptidoglycan construction *via* the Mur pathway [47]. This second putative toxin may be a new type of T6SS effector targeting substrates for peptidoglycan construction, although this remains to be confirmed. The next ORF is homologous to *sui1*, encoding a translation initiation factor [48]. A gene encoding a putative arginine decarboxylase was found upstream from *vgrG3*. The *tec3* gene, homologous to the *tec* gene described by Liang *et al*., was identified downstream from *vgrG3*. Tec proteins (members of the DUF 4123 family) have been shown to act as chaperone proteins [49]. These results suggest that the *hcp3* cluster may be involved in the T6SS-mediated antibacterial activity of MFE01.

### Hcp3-mediated T6SS killing activity is prey strain-dependent

**Fig 2:**
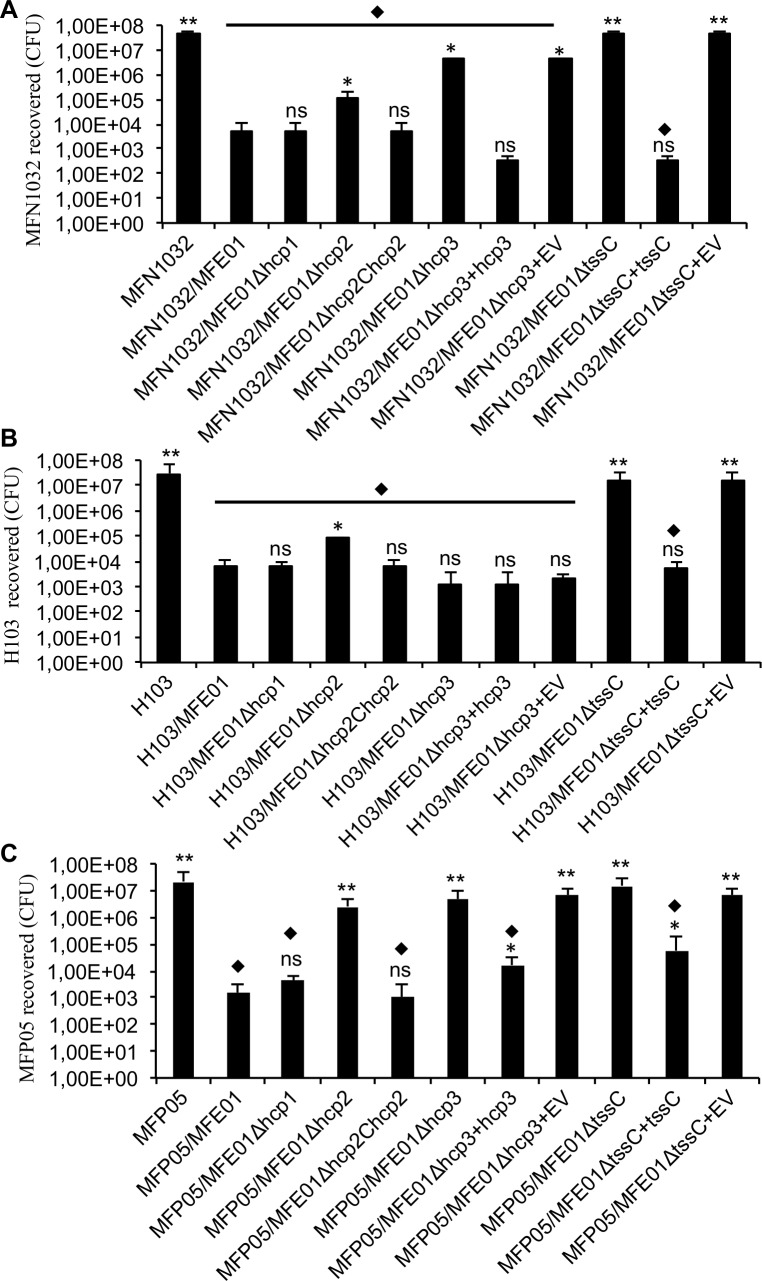
Killing activity of MFE01 and mutant strains against *P*. *fluorescens* MFN1032, *P*. *aeruginosa* H103, and *P*. *fluorescens* MFP05 during contact on solid media. Quantitative co-culture assays. **A.** Prey cells (MFN1032 carrying pSMC2.1 gfp) were cultured alone or mixed with *P*. *fluorescens* MFE01 or various MFE01 T6SS mutants in a 1:5 ratio. After incubation for 4 h at 28°C, the MFN1032 colonies were counted (*n* = 6, the error bars represent the standard error of the mean). ** indicates a significant difference in the number of MFN1032 cfu (*p*-value < 0.01) relative to MFN1032 co-cultured with MFE01; * indicates a significant difference in the number of MFN1032 cfu (*p*-value < 0.05) relative to MFN1032 co-cultured with MFE01; ns indicates no significant difference. ⧫ indicates a significant difference in the number of MFN1032 cfu (*p*-value < 0.05) relative to the MFN1032-alone control assay. EV means empty pPSV35 (plasmid control). **B.** Prey cells (*P*. *aeruginosa* H103 carrying pSMC2.1 gfp) were cultured alone or mixed with *P*. *fluorescens* MFE01 or various MFE01 T6SS mutants in a 1:5 ratio. After 4 h at 28°C, H103 colonies were counted (*n* = 6, the error bars represent the standard error of the mean). ** indicates a significant difference in the number of H103 cfu (*p*-value < 0.01) relative to H103 co-cultured with MFE01; * indicates a significant difference in the number of H103 cfu (*p*-value < 0.05) relative to H103 co-cultured with MFE01; ns indicates no significant difference. ⧫ indicates a significant difference in the number of H103 cfu (*p*-value < 0.05) relative to the H103-alone control assay. EV means empty pPSV35 (plasmid control). **C.** Prey cells (MFP05 carrying pSMC2.1 gfp) were cultured alone or mixed with *P*. *fluorescens* MFE01 or various MFE01 T6SS mutants in a 1:5 ratio. EV indicates empty vector for the pPSV35 control. After 4 h at 28°C, MFP05 colonies were counted (*n* = 6, the error bars represent the standard error of the mean). ** indicates a significant difference in the number of MFP05 cfu (*p*-value < 0.01) relative to MFE05 co-cultured with MFE01; * indicates a significant difference in the number of MFE05 cfu (*p*-value < 0.05) relative to MFE05 co-cultured with MFE01; ns indicates no significant difference. ⧫ indicates a significant difference in the number of MFE05 cfu (*p*-value < 0.05) relative to the MFE05-alone control assay.

We investigated the contribution of Hcp3 to competitor killing by co-culturing MFE01 or various MFE01 T6SS mutants and prey cells on a filter on solid medium for 4 h at 28°C. The strain MFN1032 is a clinical isolate of *Pseudomonas fluorescens* [50]. The population of *P*. *fluoresc*ens MFN1032 prey cells was significantly smaller (4 log) when cultured with MFE01 than when cultured alone (Fig 2A). The size of the MFN1032 population did not differ significantly between co-cultures with MFE01 and co-cultures with MFE01*Δhcp1*. The MFE01*Δhcp2* mutant had significantly weaker antibacterial activity than MFE01 and MFE01*Δhcp1*. We detected no significant killing during co-culture with MFE01*ΔtssC*, indicating that the T6SS was responsible for the observed antibacterial activity. The MFE01*Δhcp3* mutant was less bactericidal than MFE01, but nevertheless significantly reduced the MFN1032 population. Hcp3 is thus responsible for some of the T6SS-mediated killing activities of MFE01 against MFN1032.

We investigated the predation of MFE01 on *Pseudomonas aeruginosa* H103 (Fig 2B) [51]. The results were similar to those obtained for MFN1032, except for co-culture with MFE01*Δhcp3*. The MFE01*Δhcp3* mutant had a similar level of killing activity to the wild-type MFE01 strain. This result suggests that Hcp3 is not required for the killing of H103 mediated by the T6SS of MFE01.

In our previous studies, *P*. *fluorescens* strain MFP05, isolated from human skin, was killed on a solid surface by MFE01, and was the strain giving the highest assay sensitivity [36,52]. We studied the effect of *tssC* and *hcp3* mutations on this predation (Fig 2C). These mutations totally abolished MFE01 killing activity, which was partially restored by the reintroduction of *tssC* or *hcp3*. Thus, Hcp3 may be critical for the MFE01 T6SS-mediated killing of MFP05.

**Fig 3:**
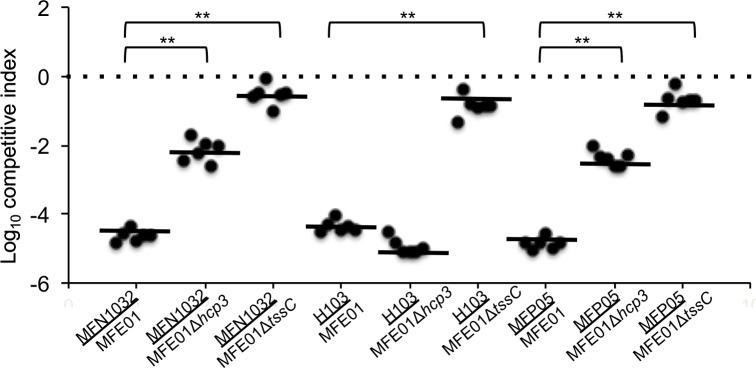
Competitive index of MFE01, MFE01Δt*ssC and M*FE01Δ*hcp3*. MFE01, MFE01Δt*ssC or M*FE01Δ*hcp3* (attackers) were mixed with MFP05, MFN1032 or H103 (preys). The mixture was incubated on nutrient agar for 4 h, and survival were enumerated by plating survivors on appropriate selective plates. The competitive index is calculated using the equation: (input attacker/input prey)/(output attacker/output prey). Horizontal bars indicate the arithmetic mean of log-transformed data. ** indicates statistical significance (Wilcoxon signed rank test, p-value < 0.05), n = 6.

The involvement of T6SS in the killing activity was confirmed by analysis of competitive index (Fig 3). The MFE01 strain outcompeted MFP05, H103 and MFN1032 (log_10_ competitive index around -5). On the contrary, the MFE01*ΔtssC* mutant was unable to outcompeted the three prey strains (log_10_ competitive index around 0). Competitive index of MFE01*Δhcp3* indicated that Hcp3 is not responsible for the MFE01 T6SS-mediated killing of H103, in contrast to that was obtained for MFN1032 and MFP05.

### MFE01 abolishes the formation of biofilms by competing bacteria during co-inoculation

**Fig 4:**
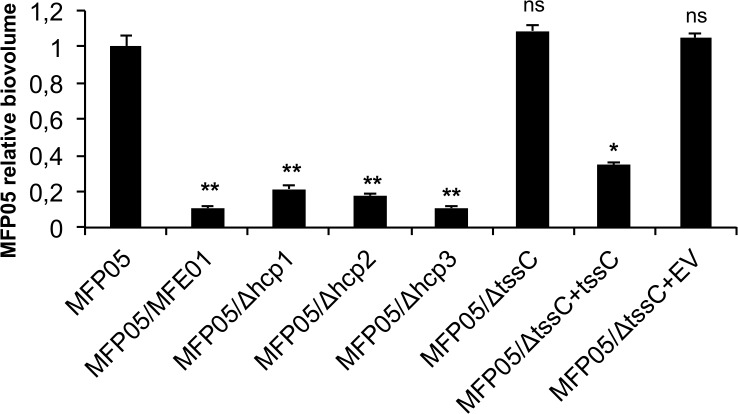
Effect of MFE01 and T6SS mutants on MFP05 biofilm formation. Biofilms were grown on a glass surface, for 48 h at 28°C, under a flow of LB medium. Biovolumes of fluorescent bacteria were determined by COMSTAT analysis after confocal laser scanning microscopy observation. *P*. *fluorescens* MFP05 bearing pSMC2.1 *gfp*, encoding green fluorescent protein, was co-cultured alone or with MFE01 or derivatives, in a 1:5 ratio. Each histogram represents the biovolume of fluorescent MFP05, relative to that of fluorescent MFP05 when MFP05-*gfp* is cultivated alone. Comparisons were made with the control MFP05-*gfp*; ** *p*-value < 0.01; * *p*-value < 0.05; ns = non-significant; *n* = 6 (the error bars represent the standard error of the mean).

During the first step of biofilm formation, bacteria must adhere to a solid surface [53]. MFE01 may kill competitor bacteria in two-species biofilms through the action of the T6SS during this adhesion step. We tested this hypothesis by incubating MFE01, MFE01Δ*hcp1*, MFE01Δ*hcp2*, MFE01Δ*hcp3*, *or* MFE01*ΔtssC* with MFP05 on a glass surface in flow chambers. Contact with MFE01 strongly reduced the biovolume of the MFP05 biofilm. By contrast, co-inoculation with MFE01*ΔtssC* did not affect MFP05 biofilm biovolume, which was similar to that obtained with MFP05 alone (Fig 4). Mutation of *hcp1*, *hcp2*, or *hcp3* did not significantly modify the reduction in MFP05 biofilm biovolume relative to co-inoculation with the wild-type MFE01 strain. These results suggest that the T6SS is involved in the decrease in MFP05 biofilm biovolume, but that none of the three *hcp* genes is indispensable for this competition. This result also suggests that another factor leading to antibacterial activity is activated only under biofilm conditions.

A similar inhibition of biofilm formation has already been described for *Pseudoalteromonas*, which was found to predominate over strains of *Paracoccus* sp. or *Vibrio* sp. in two-species biofilms. The supernatant of the *Pseudoalteromonas* liquid culture was devoid of antibacterial activity against free-living *Paracoccus* and *Vibrio* cells, but it impaired the ability of these species to grow as single-species biofilms. It also impaired biofilm formation by *Pseudomonas aeruginosa* [40]. We treated MFP05 with MFE01 supernatant during the adhesion step of the biofilm experiment in flow chambers to determine whether exoproducts secreted by MFE01 inhibited biofilm formation. The MFE01 supernatant had no significant effect on MFP05 biofilm formation (S1 Fig).

The T6SS-mediated inhibition of biofilm formation has been described in two-species biofilms containing *Burkholderia thailandensis* [54]. The threonine phosphorylation pathway (TPP) of *P*. *aeruginosa* activates the *P*. *aeruginosa* H1-T6SS cluster, which is involved in antibacterial activity, during culture on a solid surface [55]. The Gac/Rsm pathway upregulates H1-T6SS and biofilm formation, but downregulates H2-T6SS and H3-T6SS [24]. The tight regulation of the T6SS suggests that all *hcp* clusters may be regulated under biofilm conditions, working together to inhibit the formation of biofilms by other species.

### T6SS is involved in the structure of MFE01 biofilms

**Fig 5:**
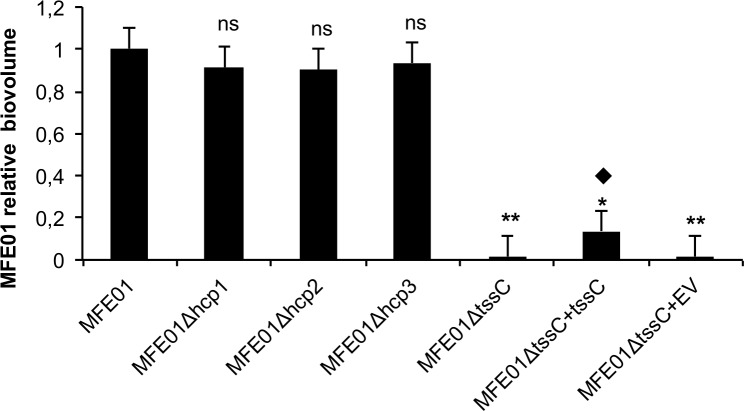
Effects of *hcp and tssC gene* mutations on biofilm biovolume in *P*. *fluorescens* strain MFE01. Biofilms were grown on a glass surface, for 48 h at 28°C, under a flow of LB medium. Bacteria were visualised with the Syto 9® green fluorescent nucleic acid stain. Biovolumes were determined by COMSTAT analysis, after confocal laser scanning microscopy observation. The values shown are biofilm biovolumes relative to that of wild-type MFE01. The data presented are the mean values for at least five independent experiments and the error bars represent the standard error of the mean. Statistical analyses were performed with non-parametric Mann-Whitney tests (two-tailed): ns indicates no significant difference in biovolume (*p*-value > 0.05) relative to the MFE01 biofilm, * and ** indicates significant difference in biovolume relative to the MFE01 biofilm: *p-value < 0.05, ** *p*-value < 0.01. ⧫ indicates a significant difference in biovolume (*p*-value < 0.05) relative to the MFE01Δ*tssC* biofilm. EV means empty pPSV35 (plasmid control).

Polysaccharides are an important component of the biofilm matrix [56]. We have already shown that the *hcp1* mutation in *P*. *fluorescens* MFE01 halves exopolysaccharide (EPS) accumulation and impairs motility (with an absence of flagella), whereas the mutation of *hcp2* does not [36]. We assessed the capacity of MFE01 mutants to form a biofilm in flow cell chambers, to determine whether these characteristics, associated with *hcp1* gene expression, influenced biofilm formation. There was no significant difference in biofilm biovolume between MFE01Δ*hcp1*, MFE01Δ*hcp2*, or MFE01Δ*hcp3* and the wild-type MFE01 (Fig 5). MFE01Δ*hcp1* retained the ability to form a biofilm, despite the decrease in EPS accumulation and the absence of flagella. This absence of flagella remains unexplained and is under investigation. Flagella do not seem to be essential for the adhesion of MFE01, and the smaller amount of EPS produced by MFE01Δ*hcp1* was still sufficient to permit adhesion.

Surprisingly, the biofilm biovolume of MFE01Δ*tssC* was significantly smaller than that of wild-type MFE01 (by a factor of 20). However, the wild-type MFE01 and MFE01Δ*tssc* growth curves were similar, indicating that the *tssC* mutation had no effect on growth kinetics. The reported impacts of T6SS gene cluster mutations on biofilm formation are diverse. In APEC (avian-pathogenic *Escherichia coli*), a mutation of the gene encoding IcmF (TssM), a structural protein of the T6SS, leads to a loss of adhesion to HeLa cells. This T6SS mutant also displays defective biofilm formation on glass [30]. T6SS mutants of *Acidovorax citrulli* also display poor biofilm formation [57]. Conversely, a deletion of *tssM*, encoding the T6SS structural protein TssM in *Acinetobacter baumanii*, reduces Hcp secretion, but does not alter biofilm formation [58]. In *Vibrio alginolyticus*, the phosphatase PppA, encoded by one of the genes of the T6SS gene cluster, downregulates *hcp* gene expression and biofilm formation [59]. Finally, a deletion in *icmF3* (*tssM*-like gene) in *P*. *aeruginosa* has been shown to enhance biofilm formation [60]. These studies show that the T6SS can affect biofilm formation, but that its role may depend on context.

**Fig 6:**
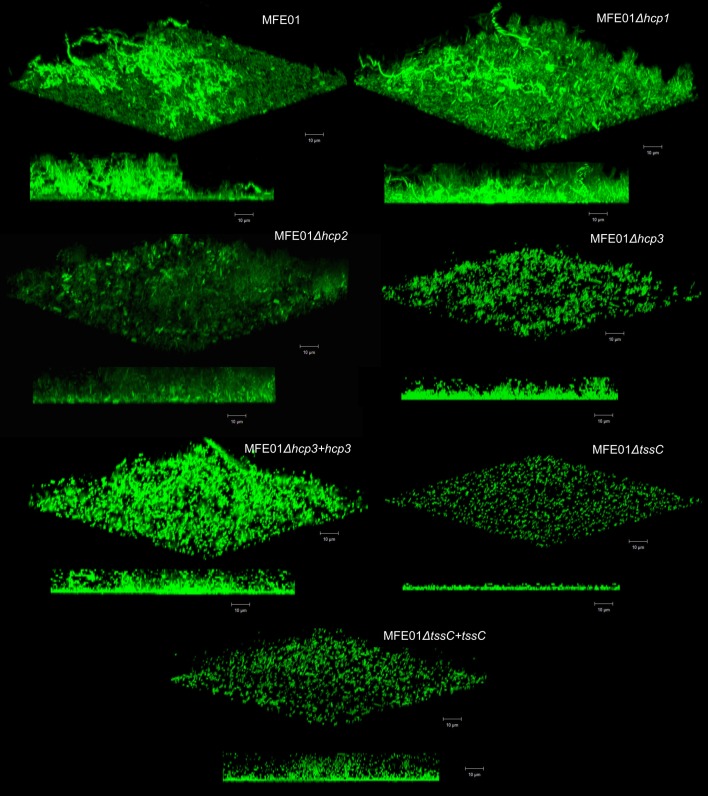
Effect of *hcp and tssC* gene mutations on the maturation of *P*. *fluorescens* MFE01 biofilms. Biofilms were grown on a glass surface for 48 h at 28°C, under a flow of LB medium. Bacteria were visualised with the Syto 9® green fluorescent nucleic acid stain. A 3D shadow representation and a side-view projection are shown at the top and bottom, respectively, for each strain. Images show representative data from at least five independent biofilm assays. Bars, 10 μm.

The measurement of biofilm biovolume provides information about the amount of biofilm, but not its level of maturation. We therefore observed cross-sections of biofilm from MFE01 or the mutants by confocal microscopy, to assess the structure of the biofilm and its degree of maturation (Fig 6). Separately, the mutations of individual *hcp* genes did not abolish biofilm formation. MFE01 biofilms were heterogeneous in appearance, with a slightly hairy surface, and contained cell aggregates but no mushroom-like structures. Although not flat, the MFE01*Δhc*p1 biofilms were more homogeneous, with less evident cell aggregates. MFE01*Δhcp2* biofilms did not have a hairy surface, but they contained early mushroom-like structures. MFE01*Δhc*p*3* biofilms were flat, homogeneous, and thicker. However, the loss of biofilm structure did not lead to a loss of biomass. Indeed, the biofilms produced by the mutant strains appeared to be more compact than the MFE01 biofilm, possibly explaining the lack of decrease in the biovolumes of the biofilms in the mutant strains. These results indicate that the expression of *hcp1*, *hcp2*, and *hcp3* influences the maturation of the biofilm, but not its biovolume. The expression of these genes is required for the formation of a mature biofilm, suggesting synergy between the three *hcp* clusters in the maturation process. These results show that none of the secreted T6SS proteins considered, Hcp1, Hcp2, or Hcp3, is the major factor involved in biofilm formation. The MFE01Δ*tssC* mutant displayed the lowest level of biofilm maturation and the greatest decrease in biofilm biovolume. Our results demonstrate that the adhesion step is unchanged in the mutant strains, contrary to the biofilm maturation.

### MFE01 may lack the conventional *Pseudomonas* quorum-sensing pathway

**Fig 7:**
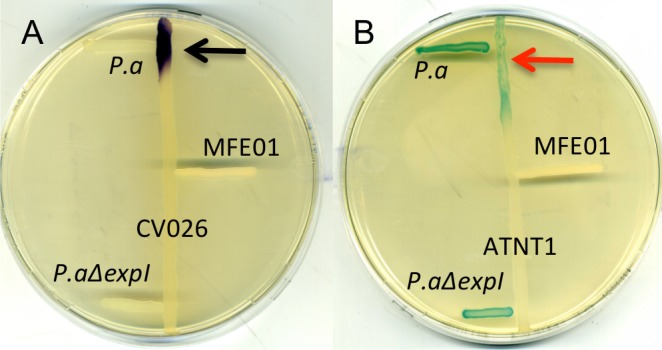
Detection of AHLs with biosensor strains. *P*.*a* indicates *Pectobacterium atrosepticum 6276* strain, which produces C_8-_ NAHLs, used as positive control. *P*.*aΔexp1* means *Pectobacterium atrosepticum* 6276 mutant strain, which does not produce AHLs, used as negative control. **A.** Detection of short-chain AHLs with *Chromobacterium violaceum* CV026 (CV026). CV026 indicates *C*.*violaceum CV026* strain, which produces vioalacein in contact with C_4_-C_8_ NAHLs, used as biosensor strain. Black arrow indicates violacein production by *C*.*violaceum CV026* strain. LB plates were incubated for 72 h at 37°C (*n* = 3). **B.** Detection of long-chain AHLs with *Agrobacterium tumefaciens* NTI (ANTI1). ANT1 means *Agrobacterium tumefaciens* NT1 strain, which produces β-galactosidase in contact with C_6_-C_12_ NAHLs, used as biosensor strain. Red arrow indicates β-galactosidase production by *Agrobacterium tumefaciens* strain. LB plates containing X-Gal (40 μg/mL) were incubated for 72 h at 28°C (*n* = 3).

Numerous factors regulate biofilm formation. In *P*. *aeruginosa*, biofilm formation and T6SS expression are linked to chronic infection and are highly regulated. The sensor RetS downregulates these phenotypes *via* c-di-GMP signalling [61]. Biofilm formation and T6SS expression in *Burkolderia cenocepacia* are upregulated by quorum sensing and downregulated by the sensor kinase AtsR [62]. Many studies in *P*. *fluorescens* have described the regulation of biofilm formation through quorum sensing (QS), biosurfactants, and the C-di-GMP or Gac/Rsm pathway [25]. Contrary to *Pseudomonas aeruginosa* and *Pseudomonas putida* species, *Pseudomonas ffuorescens* species seems devoid of quinolone-based QS system [63]. As described by Martins *et al*. [64], quorum sensing systems based on N-acyl-homoserine lactone (AHL) signalling molecules have been identified in a few *P*. *fluorescens* strains [37,65–68]. Several studies have shown that other strains of *P*. *fluorescens* do not produce AHLs [64,69,70]. Standalone blast analyses of the MFE01 draft genome indicated that it did not contain any genes encoding a protein corresponding to the conserved AHL synthase (WP_044464955.1). We screened for AHL production, using biosensor strains to verify the absence of AHL synthesis. *Pectobacterium atrosepticum 6276* and *Pectobacterium atrosepticum 6276 ΔexpI* were used as positive and negative controls for AHL production, respectively [71]. We screened for short-chain AHL production with the biosensor *Chromobacterium* violaceum CV026. This biosensor responds to C4-C8 AHLs by producing a purple pigment (violacein) after overnight incubation at 28°C. No purple pigmentation was observed around CV026 streak when MFE01 was cross-streaked with CV026 (Fig 7A). We screened for AHL production with *Agrobacterium tumefaciens* NTI. This biosensor responds to C6-C12 AHLs by producing ß-galactosidase, the activity of which generates a blue colour on X-gal plates after overnight incubation at 28°C. No blue colour was observed around *Agrobacterium tumefaciens* NTI streak after overnight incubation following cross-streaking with MFE01 (Fig 7B). This preliminary screening indicates that, like many other *P*. *fluorescens* strains, MFE01 does not produce short- or long-chain AHLs recognised by the two used biosensors. The T6SS system may act as a quorum-sensing pathway in MFE01. Vettiger *et al*. demonstrated that T6SS substrates (*i*.*e*. Hcp, VgrG, and effectors) were transferred between and reused in sister cells [71]. The amount of T6SS substrate received by a cell depends on cell density and seems to follow the same principle as the auto-inducer accumulation mechanism responsible for quorum sensing.

## Conclusion

We describe here several characteristics of the T6SS of the environmental *P*. *fluorescens* strain MFE01. We show that Hcp1, Hcp2, and Hcp3 may be involved in T6SS-mediated competition with other *Pseudomonas* strains on solid media, depending on the prey cell. MFE01 also impaired the formation of biofilms by *P*. *fluorescens* MFP05, a strain isolated from skin. Our findings highlight the potential of MFE01 as a biocontrol agent for preventing biofilm formation by another *Pseudomonas* strain isolated from skin. The protective barrier provided by human skin can be breached by burn wounds, which render the patient susceptible to bacterial infection. *P*. *aeruginosa* is frequently isolated from burn patients and causes serious infection [72,73], accounting for more than 50% of all deaths of these patients from infection [74]. Infections with multidrug-resistant strains of *P*. *aeruginosa* may also be untreatable with antibiotics, highlighting the need to develop new therapies [75]. For this reason, the ability of *Lactobacillus* to inhibit the growth of harmful bacteria in burn wounds has been investigated [76].

Although the inhibition of biofilm formation was related to T6SS integrity, we were unable to demonstrate a predominant role for any of the three Hcp proteins. Moreover, a mutant defective in the *tss*C gene of the unique T6SS core component could not inhibit biofilm formation or produce a biofilm under our conditions. We hypothesise that T6SS effectors may be used as a cell-to-cell signal among sister cells in this strain, which seems to be devoid of the classical AHL quorum-sensing pathway.

## Supporting Information

S1 Fig Effect of MFE01 and T6SS mutants on MFP05 biofilm formation. To investigate the effects of *MFE01* exoproducts on biofilm formation by MFEP05, MFP05-*gfp* bacteria, bearing pSMC2.1 *gfp*, were diluted to an OD580 of 0.25 in a supernatant of MFE01. This supernatant is obtained from a liquid culture of MFE01 grown overnight that was sterilized by filtration through a membrane with pores with a 0.22-μm diameter. MFP05-*gfp* bacteria diluted in supernatant of MFE01 were then inoculated in flow cell channels, and the 2-h adhesion step under static conditions followed by biofilm growth under a flow of fresh LB medium was performed for 48 h at 28°C. Biovolumes of fluorescent bacteria were determined by COMSTAT analysis after confocal laser scanning microscopy observation. MFP05+S histogram represents the biovolume of fluorescent MFP05 exposed to the supernatant of MFE01, relative to that of fluorescent MFP05 when MFP05-*gfp* is diluted in LB.*ns = non-significant; *n* = 6 (the error bars represent the standard error of the mean).
(PDF)
