# Supplementary material for: Contribution of the Pseudomonas fluorescens MFE01 Type VI Secretion System to Biofilm Formation
Source: PLoS One. 2017 Jan 23;12(1):e0170770. doi: 10.1371/journal.pone.0170770 (PMC5256989; doi:10.1371/journal.pone.0170770)
Supplement: S1 Fig — To investigate the effects of MFE01 exoproducts on biofilm formation by MFEP05, MFP05-gfp bacteria, bearing pSMC2.1 gfp, were diluted to an OD580 of 0.25 in a supernatant of MFE01. This supernatant is obtained from a liquid culture of MFE01 grown overnight that was sterilized by filtration through a membrane with pores with a 0.22-μm diameter. MFP05-gfp bacteria diluted in supernatant of MFE01 were then inoculated in flow cell channels, and the 2-h adhesion step under static conditions followed by biofilm growth under a flow of fresh LB medium was performed for 48 h at 28°C. Biovolumes of fluorescent bacteria were determined by COMSTAT analysis after confocal laser scanning microscopy observation. MFP05+S histogram represents the biovolume of fluorescent MFP05 exposed to the supernatant of MFE01, relative to that of fluorescent MFP05 when MFP05-gfp is diluted in LB.*ns = non-significant; n = 6 (the error bars represent the standard error of the mean). (PDF) [file pone.0170770.s001.pdf]

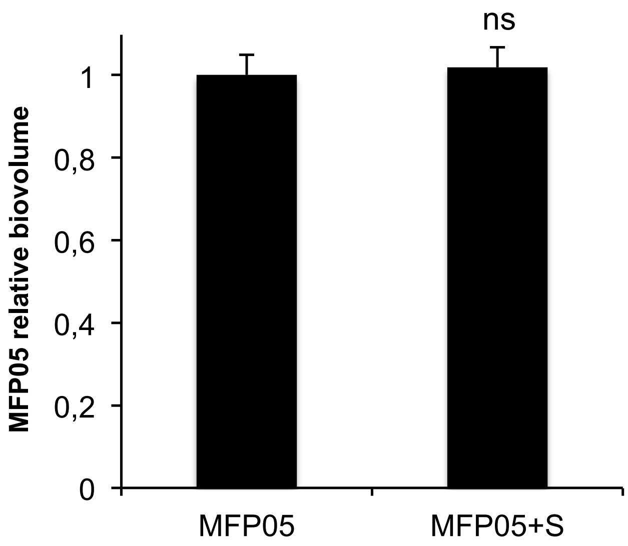

S1 figure. Effect of MFE01 and T6SS mutants on MFP05 biofilm formation

To investigate the effects of *MFE01* exoproducts on biofilm formation by MFEP05, MFP05-*gfp* bacteria, bearing pSMC2.1 *gfp*, were diluted to an OD580 of 0.25 in a supernatant of MFE01. This supernatant is obtained from a liquid culture of MFE01 grown overnight that was sterilized by filtration through a membrane with pores with a 0.22-μm diameter. MFP05-*gfp* bacteria diluted in supernatant of MFE01 were then inoculated in flow cell channels, and the 2-h adhesion step under static conditions followed by biofilm growth under a flow of fresh LB medium was performed for 48 h at 28°C. Biovolumes of fluorescent bacteria were determined by COMSTAT analysis after confocal laser scanning microscopy observation. MFP05+S histogram represents the biovolume of fluorescent MFP05 exposed to the supernatant of MFE01, relative to that of fluorescent MFP05 when MFP05-*gfp* is diluted in LB.*ns = non-significant; *n* = 6 (the error bars represent the standard error of the mean).
